# CT-generated radiographs in patients with pelvic ring injury: can they be used in lieu of plain radiographs?

**DOI:** 10.1186/s13018-016-0361-6

**Published:** 2016-02-22

**Authors:** Adham A. Abdelfattah, Berton R. Moed

**Affiliations:** Department of Orthopaedic Surgery, Saint Louis University School of Medicine, 3635 Vista Avenue, 7th Floor Desloge Towers, St. Louis, MO 63110 USA; The Hansjörg Wyss Endowed Chair in Orthopaedic Surgery, Department of Orthopaedic Surgery, Saint Louis University School of Medicine, 3635 Vista Avenue, 7th Floor Desloge Towers, St. Louis, MO 63110 USA

**Keywords:** Pelvic ring injury, CT-generated radiographs, Pelvic fractures

## Abstract

**Background:**

Pelvic ring injury classification traditionally is made using plain radiographs. Recent studies suggest that computed tomography (CT)-generated images have higher diagnostic accuracy than plain films for the classification of acetabular fractures. However, similar studies have not been performed for pelvic ring injuries. The purpose of this study was to compare CT-generated and plain radiographs in terms of the ability of surgeons at different experience levels to identify pelvic injury type.

**Methods:**

CT-generated and plain radiograph image sets were created from 15 pelvic ring injury patients with known classification morphology. Three groups, each consisting of three orthopaedic surgeons representing different levels of expertise, viewed these image sets and recorded their diagnoses. These diagnoses were compared to the gold standard findings of the treating physician and to each other.

**Results:**

Overall, there was a significantly improved ability to correctly classify pelvic ring injury type by CT-generated radiographs as compared to plain radiographs (*p* < 0.01). However, analysis of the groups revealed that this difference was limited to the less experienced groups (*p* < 0.05).

**Conclusions:**

CT-generated radiographs are diagnostically beneficial for less experienced surgeons and at least as good as conventional plain radiographs for experienced surgeons in classifying pelvic ring injuries. Therefore, CT-generated radiographs may be clinically valuable: sparing the patient additional radiation exposure and discomfort by avoiding the reordering of plain radiographs when the initial studies are of poor quality, as well as serving as a possible alternative for supplemental initial injury plain radiographic views.

## Background

Classification of disruptions of the pelvic ring is important not only in the determination of initial patient management but also because it provides prognostic data and the information needed to plan definitive care [1–9]. The classification schemes of Young and Burgess [3] and Tile [4] described the severity of injury by the mechanistic process involved and by grading the stability of the injury, respectively. However, the most current classification method is that developed by the AO and the Orthopaedic Trauma Association (OTA) and is a composite of these two earlier methods [5].

Currently, the classification of a pelvic ring injury is based on evaluation of conventional plain radiographs, specifically the anteroposterior (AP), inlet and outlet views [3, 4, 7, 8, 10]. Although plain radiographs have been the mainstay of classifying pelvic ring disruptions, plain radiographs are subject to factors, such as obesity and the presence of bowel gas or contrast media, that can impair diagnostic accuracy [11–13]. In addition, accurate positioning of the X-ray tube angle to obtain proper views of the pelvis may be difficult to achieve, depending on the patient’s condition and the technician’s precision in obtaining these films [10, 11]. Recent computed tomography (CT) advancements allow the creation of two-dimensional images that approximate AP and oblique plain radiograph views, using a standardized methodology and widely available commercial software computer workstation software, with the CT data obtained as part of the standard trauma evaluation [14, 15]. Since these unshaded volume rendered CT-generated (CT-G) images are created from the CT scan which is obtained as part of the standard trauma evaluation, the patient does not incur any additional radiation exposure [14]. Recent studies suggest that these CT-G images have higher diagnostic accuracy than plain films for the classification of fractures of the acetabulum [16, 17]. However, similar studies have not been performed for the assessment of pelvic ring injuries. The purpose of this study was to compare CT-G and plain radiographs in terms of the ability of surgeons at different experience levels to identify pelvic injury type.

## Methods

**Fig. 1:**
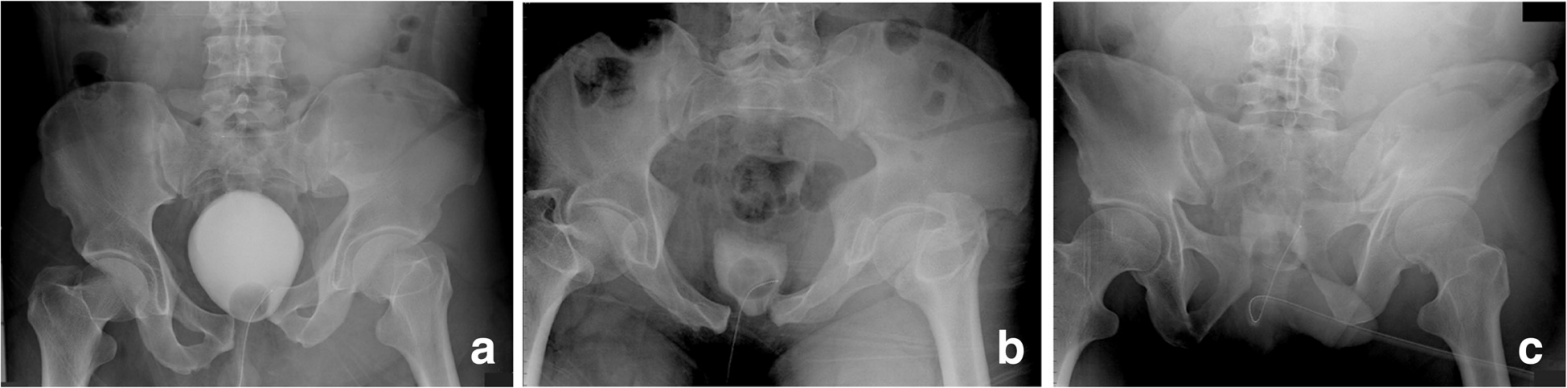
Conventional plain AP (**a**), inlet (**b**) and outlet (**c**) pelvic radiographs obtained on the day of injury in a polytrauma patient

**Fig. 2:**
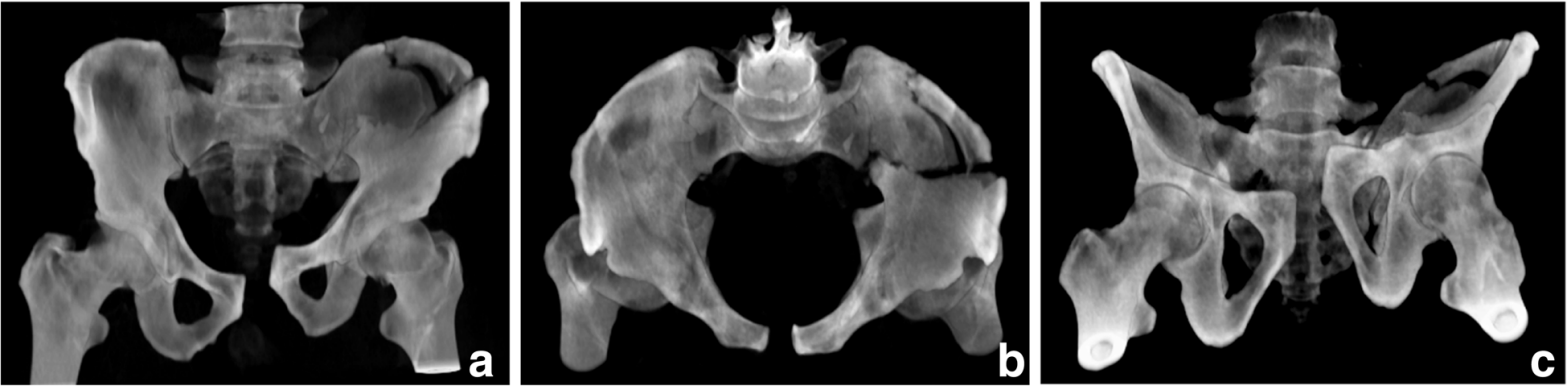
CT-generated AP (**a**), inlet (**b**) and outlet (**c**) pelvic radiographs of the patient from Fig. 1

After obtaining Institutional Review Board approval, we retrospectively reviewed a database of all trauma patients presenting to our level 1 trauma centre from June 2009 to July 2011 with pelvic ring injuries. This was performed in accordance with the Declaration of Helsinki and was approved by our ethics committee (The Saint Louis University Institutional Review Board, Protocol Number: 17190). Two image sets were created from patients, representing the spectrum of pelvic ring injury, whom had a complete series of plain pelvic radiographs (AP, inlet and outlet) and a pelvic CT scan at time of their initial presentation to the hospital as a routine part of their acute evaluation. We excluded patients who had been stabilized using a pelvic compression device, such as a pelvic binder, which was in place at the time of imaging. Set A consisted of AP, inlet and outlet conventional plain radiographs (Fig. 1a–c) and set B consisted of AP, inlet and outlet CT-G radiographs (Fig. 2a–c). All plain radiographs and CT studies were obtained upon the patients’ presentation to the emergency department as part of their routine medical care. All CT scans were obtained with 1- to 3-mm slice sections using a Seimens Somatom 40 detector CT scanner (Siemens AG, Berlin and Munchen, Germany). The CT-G radiographs were created by trained radiology technicians using standardized methodology, computer workstation and software (SyngoMMWP, Siemens AG, Berlin and Munchen, 2010). Therefore, a total of 30 image sets were created for evaluation. Images were then de-identified and assigned numbers for randomization using a random number generator (Microsoft Office Excel 2003; Microsoft Corporation, Redmond, WA). They were then randomly arranged in an alternating plain and CT-G radiograph order. The images were transferred from Synapse^®^ picture archiving and communication system (FUJIFILM Medical Systems USA, Inc., Stamford, CT, USA) and presented to the study group as a Microsoft^®^ PowerPoint^®^ (Microsoft Corp) presentation.

Three study participant observer groups were created for comparison based on the level of training. These included an attending level (three fellowship-trained orthopaedic traumatologists), a senior resident level (three postgraduate year 4 and 5 residents) and a junior resident level (three postgraduate year 2 and 3 residents). The method for classifying the pelvic ring injury was reviewed with each participant [5]. Each participant independently analysed the same series of 30 image sets and each recorded their diagnosis using the Orthopaedic Trauma Association classification system [5]. In this way, the injuries were classified by the main category as type 61-A (a lesion sparing or with no displacement of the posterior arch), type 61-B (incomplete disruption of the posterior arch; partially stable) or type 61-C (complete disruption of the posterior arch; unstable) [5]. In addition, they were further classified into one of the three subgroups for each main category [5]. Their diagnoses were compared to the gold standard findings as determined by the treating surgeon, who was not a member of the study group and used all available imaging studies (including the two-dimensional CT data), as well as the findings at the time of any surgical intervention (Table 1). Proportional probability statistics (Z-scores) were used to evaluate differences in surgeon performance using CT-G and plain radiographs. Statistical analyses were performed using a commercially available statistical package with alpha set at 0.05. A priori sample size calculations could not be performed. However, post hoc sample size calculation with the 45 observations by 3 raters in each training level indicated 80 % power to detect a 16-point difference in the percentage of correctly classified fractures, with alpha set at 0.05.

**Table 1:** Gold standard pelvic ring injury type

OTA fracture type	Number in study
Type 61-A 1	2
Type 61-A 2	1
Type 61-A 3	0
Type 61-B 1	1
Type 61-B 2	6
Type 61-B 3	0
Type 61-C 1	2
Type 61-C 2	1
Type 61-C 3	2

As determined by the treating surgeon

## Results

Overall, considering all subject groups together (Table 2), the CT-G radiographs were statistically better than the plain radiographs for the accurate classification of the pelvic ring injury. However, as experience level decreased, so did performance using the plain radiographs. While the performance of the trauma-trained attending faculty was not significantly different using the plain radiographs as compared to CT-G radiographs in correctly classifying the pelvic fractures (Table 2), both the senior and junior resident groups performed significantly worse using the plain radiographs. Senior residents correctly identified the pelvic fracture in 51 % using conventional plain radiographs as compared to 73 % when using CT-G images (Table 2). The junior residents correctly identified the pelvic fracture in only 31 % using conventional plain radiographs as compared to 53 % using CT-G radiographs (Table 2).

**Table 2:** Performance for the two evaluating methods as compared to the gold standard

Experience level group	Agreement with gold standard (number of observations)		
	Plain radiographs	CT-generated radiographs	*p* value
All observers	63/135 (47 %)	90/135 (67 %)	<0.01
Trauma attendings	26/45 (58 %)	33/45 (73 %)	0.12
Senior residents	23/45 (51 %)	33/45 (73 %)	0.03
Junior residents	14/45 (31 %)	24/45 (53 %)	0.03

Comparison between the groups by experience level showed that the senior residents’ responses were not significantly different from those of the trauma-trained attending faculty (Table 3). However, the junior residents had less accurately identified the correct pelvic ring injury classification as compared to the trauma-trained attending faculty and senior residents, no matter what imaging modality was used (Table 3). A post hoc calculation determined that this study had a power of 92 % to detect the observed difference in the gold standard agreement between plain and CT-G radiographs across all 135 observations made by the 9 raters with alpha set at 0.05.

**Table 3:** Comparative analysis of performance between groups

Observer group	Radiograph type	
	Plain	CT-generated
Trauma-trained faculty vs. junior residents	*p* < 0.05	*p* < 0.05
Junior vs. senior residents	*p* < 0.05	*p* < 0.05
Trauma-trained faculty vs. senior residents	*p* = 0.53	*p* = 1.00

## Discussion

Identifying the correct type of pelvic ring injury is critical in directing the appropriate treatment to reduce morbidity and mortality [1, 2]. Good patient outcomes of pelvic fractures are dependent on timely recognition and intervention that is based on an accurate diagnosis. Historically, the Young and Burgess [3] and Tile [4] classification schemes have been used to describe these fractures. More recently, the Orthopaedic Trauma Association classification system, which is a composite of these two earlier methods, has been adopted [5]. The initial diagnosis of pelvic ring injury, as well as provisional classification, often can be made using a conventional AP radiograph obtained in the emergency room [11]. In conjunction with a two-dimensional CT scan, the conventional AP radiograph has been shown to have identified 96 % of the injured structures [11]. In any case, complete definition of the pelvic injury and the subsequent classification of these injuries are based on the evaluation of the pelvis using the inlet and outlet plain radiographic views in addition to the two-dimensional CT scan and the plain AP radiograph [4, 7, 8, 10]. Recent CT advancements that allow the creation of two-dimensional images that approximate plain radiograph views have proved effective in enhancing the diagnostic efficacy of conventional plain radiographs for the diagnosis and treatment of acetabular fractures [14–17]. Our study, which we believe is the first to evaluate CT-G imaging for pelvic ring injuries, indicates that these images have potential benefits. CT-G radiographs proved very beneficial for inexperienced surgeons and at least as good as conventional plain radiographs for experienced surgeons in accurately classifying pelvic ring injuries.

As compared to the gold standard, the performance of the trauma-trained faculty was not significantly different. However, they did provide an incorrect diagnosis in 27 % using CT-G images and in 42 % using plain radiographs. Although these incorrect percentages may seem high, they are likely due to the fact that the participants were not provided the axial, two-dimensional computed tomographic images. This finding is consistent with that of other investigators. Berg et al. found that radiographs alone identified 66 % of pelvic ring injuries correctly [11]. For acetabular fractures, O’Toole et al. likewise have shown improved diagnostic accuracy when provided all imaging modalities including CT images in conjunction with plain radiographs [16].

This study did show a hierarchy in the ability to interpret these images, which is not unexpected. As noted, the performance of the trauma-trained faculty was not significantly different in comparison to the gold standard. However, both resident groups showed significantly diminished performance using the plain radiographs as compared to the CT-G images. Furthermore, the junior residents as compared to the senior residents performed worse using either imaging modality. These findings are indicative of the importance of experience level in the interpretation of pelvic ring injury radiographs, no matter how they are obtained.

There are a number of limitations to this study. First, the retrospective nature of our patient selection does not allow for prospective standardization of the radiographic methods. However, all of the radiology technicians at our institution see a high volume of pelvic fracture patients and are well trained. These images represent the best that these technologists can obtain in a trauma setting and most likely also represent the best case scenario of what can be obtained in an orthopaedic practice. As noted, these studies were obtained as part of the patient’s routine care and did not represent an additional specialized study or cause the patient additional radiation exposure. Second, the CT images were subject to the pelvic CT protocol in place at the time the patient was treated and vary from 1–3 mm in thickness. However, our study demonstrates that despite not having a specific set protocol, radiographs generated from routinely acquired CTs are not functionally different from plain radiographs. In addition, the apparent overall low percentage of correct interpretations could be viewed as a study limitation. However, as previously noted, the performance of our attending traumatologists was similar to previously reported rates when plain radiographs are used in isolation from axial CTs [11].

Finally, the act of placing the patient in the CT scanner often does alter the position of the fracture. This phenomenon was evident to a minor degree in our study (see Figs. 1a–c and 2a–c). With the patient constrained and in the supine position during the CT procedure, CT-G radiographs can possibly result in an under appreciation or misdiagnosis of an injury, which may be more apparent using plain radiographic views. Although this issue did not affect the results in our study, this finding has been noted in the clinical setting for fractures of the acetabulum [18]. This potential problem limits the usefulness of CT-G radiographs in patients maintained in a pelvic binder, and therefore to some degree in the acute polytrauma setting, for which the conventional AP radiograph in conjunction with the two-dimensional CT scan should be adequate [11]. However, CT-G images rather than plain radiographs may be more practical in certain clinical situations. Poor quality plain pelvic oblique radiographs are not uncommon (14 % in one series) [11]. Inadequate plain radiographs can be attributed to patient motion, poor patient positioning, inadequate technique, obesity, the presence of bowel gas and residual contrast material [11–13]. The need for better quality AP and/or oblique images may then result in repeat imaging and additional exposure to radiation. Furthermore, returning the patient to the X-ray suite for repeat plain radiographs or movement of the patient to insert the X-ray cassette for portable views may be very painful and without a guarantee of obtaining satisfactory images. CT-G images are modifiable on the computer workstation. Therefore, obtaining additional views at potentially alternative angles requires neither additional patient discomfort nor additional radiation exposure. Simple adjustment of images using software can provide the best views to further define and classify the injury.

## Conclusions

When comparing the use of conventional plain radiographs to CT-G radiographs among different levels of surgeons, CT-G radiographs proved diagnostically beneficial for less experienced surgeons and at least as good as conventional plain radiographs for experienced surgeons in accurately classifying pelvic ring injuries. Therefore, CT-G radiographs may be clinically valuable in both the teaching and the patient care settings: sparing the patient additional radiation exposure and discomfort by avoiding the reordering of plain radiographs when the initial studies are of poor quality, as well as serving as a possible alternative for supplemental initial injury plain radiographic views.
